# Betaine supplementation improves CrossFit performance and increases testosterone levels, but has no influence on Wingate power: randomized crossover trial

**DOI:** 10.1080/15502783.2023.2231411

**Published:** 2023-07-06

**Authors:** Emilia Zawieja, Krzysztof Durkalec-Michalski, Marcin Sadowski, Natalia Główka, Agata Chmurzynska

**Affiliations:** a Poznan University of Life Sciences, Department of Human Nutrition and Dietetics, Poznań, Poland; b Poznan University of Physical Education, Department of Sports Dietetics, Poznań, Poland; c Charles University, Department of Physiology and Biochemistry, Faculty of Physical Education and Sport, Prague, Czech Republic

**Keywords:** Betaine, CrossFit, Wingate test, testosterone, supplementation

## Abstract

**Background:**

Because betaine (BET) supplementation may improve muscular strength and endurance, it seems plausible that BET will also influence CrossFit performance (CF).

**Purpose:**

The aim of this study was to evaluate the effects of three weeks of BET supplementation on body composition, CF performance, muscle power in the Wingate anaerobic test (WAnT), and the concentrations of selected hormones. The secondary aims were to analyze the effectiveness of two different BET doses (2.5 and 5.0 g/d) and their interaction with the methylenetetrahydrofolate reductase (MTHFR) genotype.

**Methods:**

The study was designed in a double-blinded randomized cross-over fashion. Forty-three CF practitioners completed the entire study. CF performance was measured using the Fight Gone Bad (FGB) workout and muscle power was evaluated in a 30-second WAnT. Body composition was determined by air-displacement plethysmography. Blood was drawn to assess hormone concentrations. The C677T single nucleotide polymorphism (rs180113) in the *MTHFR* gene was analyzed.

**Results:**

FGB total improved with BET by 8.7 ± 13.6% (*p* < 0.001), but no significant changes were observed with placebo (– 0.4 ± 10.0%, *p* = 0.128). No changes were also observed in WAnT and body composition. After BET supplementation testosterone concentration increased by 7.0 ± 15.4% with BET (*p* = 0.046) (no change with placebo: 1.5 ± 19.6%, *p* = 0.884) but had no effect on concentrations of insulin-like growth factor or cortisol. Finally, there were no significant interactions between *MTHFR* genotype and BET dose in any outcome.

**Conclusions:**

BET supplementation may improve CF performance and increase testosterone concentration. However, there was no evidence of a difference between dosages (2.5 and 5.0 g/d) and *MTHFR* genotypes. The trial was registered on clinicaltrials.gov (NCT03702205) on 10 October 2018.

## 1. Introduction

CrossFit (CF) is a relatively new training program with the goal of optimizing all aspects of physical capacity and performance. Daily workouts include strength, gymnastic, and endurance exercises that engage both aerobic and anaerobic energy systems in the body [1]. The effects of CF training on acute and chronic adaptations are being extensively studied. So far, studies have shown that heart rate (HR) during CF workouts ranges from 54% to 98% of maximum HR, lactate concentration at the end of workout is 6–15 mmol/L, oxygen uptake is 57–66% of maximal oxygen uptake, and rating of perceived exertion is 8–9 (10-point scale) [2]. CF practitioners frequently seek nutritional aid, such as betaine (BET) supplementation, to improve CF performance and recovery [3]. BET has recently become a common ingredient in ergogenic supplements for athletes and physically active people. BET could hypothetically affect muscle strength and power. However, the results of previous studies have been inconsistent. Hoffman et al. [4] showed that two weeks of BET supplementation improved muscle endurance and the number of squat repetitions performed at 90% of peak and mean power. Pryor et al. [5] found that one week of BET ingestion improved sprint performance on a cycloergometer. In contrast, Cholewa et al. [6] showed that BET does not enhance muscular strength. However, CF performance is highly dependent on muscular endurance since popular workouts include multiple repetitions with external load (exercises like squats, push press, kettlebell swing, deadlift, snatch, or clean) [1]. Thus, based on the studies by Hoffman et al. [4] and Pryor et al. [5] it can be hypothesized that BET may also improve CF performance.

The mechanisms of BET’s potential ergogenicity remain incompletely understood. The two main roles of BET in the body are osmoregulation and methyl group donation. The latter plays an important role in homocysteine methylation to methionine, which is then converted to S-adenosylmethionine (SAM). SAM is a universal methyl group donor for many reactions in the body including gene expression and protein synthesis. In this way BET may indirectly influence several aspects of cell metabolism. BET also prevents the formation of homocysteine thiolactone by increasing homocysteine transformation. Homocysteine thiolactone promotes insulin resistance and inactivates enzymes associated with protein synthesis, which may also hamper training adaptations [6]. In addition BET may alter the concentrations of anabolic/catabolic hormones that interact with muscle protein synthesis and breakdown processes [7].

Because BET is a part of one-carbon metabolism, the effects of BET supplementation may be dependent on the entire one-carbon cycle [8]. Under normal conditions, 5-methyltetrahydrofolate (5-MTHF) is the primary cosubstrate for homocysteine remethylation to methionine [9]. 5-MTHF is generated from 5,10-methyltetrahydrofolate (5,10-MTHF) by enzyme methylenetetrahydrofolate reductase (MTHFR) [8]. People with the disadvantageous *MTHFR* genotype (the T allele of the C677T polymorphism, rs180113) have reduced MTHFR activity and may need more BET for homocysteine metabolism. This is because BET serves as an alternative methyl donor in homocysteine methylation in a reaction catalyzed by betaine-homocysteine S-methyltransferase. BET supplementation may hence be more beneficial to T-allele carriers, at least hypothetically [10]. On the other hand, subjects who use less BET for homocysteine methylation may benefit from more available BET for other processes in the body. Yet no studies have yet investigated the effects of a single nucleotide polymorphism in the *MTHFR* gene on the response to BET supplementation.

Interestingly, previous studies used a similar small daily dose of BET (2.0–2.5 g/day), while effective doses used for clinical reasons (e.g. for lowering homocysteine concentration) tend to be higher, at 4–6 g/day. It thus seems important to establish whether higher doses of BET would produce greater improvements in physical performance than the lower doses that have often been used.

To fill in the gaps in the literature, the main goal of the present study was to evaluate the effects of three weeks of BET supplementation on CF performance, on power in the Wingate anaerobic test (WAnT), and on body composition in a group of male CF practitioners. The secondary goals were to assess the effects of different doses of BET (2.5 and 5.0 g/d) and *MTHFR* genotype (rs180113) on the effectiveness of BET supplementation. We hypothesize that 5.0 g/d BET induces greater results than 2.5 g/d and that T-allele carriers in the *MTHFR* gene (rs180113) react differently than CC homozygotes.

## 2. Methods

### 2.1. Study design

The study was designed as a double-blind, randomized, placebo-controlled crossover trial. To investigate the effects of BET supplementation, 43 participants were randomly divided into two parallel groups: one (*n* = 24) ingesting 2.5 g/d BET and another (*n* = 19) ingesting 5 g/d BET. Participants in both groups received both BET supplement and placebo (PL) in a random order. The supplementation periods (BET and PL) lasted for three weeks each and were separated by a three-week washout period. The duration of the washout seems reasonable, since a previous study showed that serum betaine concentrations return to baseline at 4 days post-supplementation (6 g/d for 14 days) [11].

The participants attended four study meetings at the Department of Human Nutrition and Dietetics and the Center of Physical Culture, Poznan University of Life Sciences, Poland. The meetings were conducted before and after each supplementation period. On each study meeting day, participants were invited to two sessions: one in the morning and another in the afternoon. During the morning session, participants had their body composition measured and blood samples were collected in the fasted state. During the afternoon session, participants performed the WAnT and Fight Gone Bad (FGB) tests separated by a twenty-minute break. The participants were familiarized with the testing procedures prior to the beginning of the study. During the familiarization session the participants were instructed about the proper technique of each FGB exercise. There was no need to practice FGB because the participants were already familiar with this workout. Then the participants watched a video of WAnT with a verbal instruction. After adjusting the height of a saddle and imposing external load, the participants practiced WAnT (3 times). All the study meetings for a given participant were performed at the same time of day. The study was conducted from January 2019 to December 2020. All athletes stated that they had not introduced any changes to their lifestyles, training, nutrition, or supplementation during the study.

Randomization of the participants was conducted using https://www.studyrandomizer.com/ with permuted block algorithm. There were four blocks (2.5 g/d BET-PL, 2.5 g/d PL-BET, 5.0 g/d BET-PL, 5.0 g/d PL-BET) with equal allocation. The randomization process and supplement preparation were conducted by the third party not engaged in the experiment and were revealed only after the study had ended to ensure concealment of the allocation sequence.

The study was approved by the local ethical committee (Bioethics Committee at Poznan University of Medical Sciences, Poznan, Poland. Decision no. 1092/17, 9 November 2017) and written informed consent was obtained from all participants before the study began. All procedures were conducted in accordance with the ethical standards of the 1964 Helsinki Declaration. The trial was registered on clinicaltrials.gov (NCT03702205) before the beginning of the study.

### 2.2. Participants

Fifty-five participants were initially enrolled to participate in this study. A total of 43 completed the entire study protocol and were included in analyses. The participants recreationally and regularly trained in CF at different gyms in Poznan, Poland. The criteria for qualifying for the study included good health, age between 18 and 45, male gender, at least one year of CF training experience, and a minimum of two CF sessions a week. The exclusion criteria were current injury or a serious injury in the six months before the study, the use of illegal performance enhancing drugs, vitamin B, choline, or BET supplementation during three weeks preceding the study, metabolic or other chronic diseases. The sample size calculation was performed using G*Power 3.1.9.7. It was estimated that a total of 26 participants were necessary to achieve an anticipated effect size = 0.30 and power (1−ß) = 0.95 at α = 0.05 with correlation among measurements = 0.5 in FGB.

### 2.3. Supplementation

Participants were randomly allocated to a group receiving either 2.5 or 5.0 g/d BET. BET was administered in the form of cellulose capsules (Medicaline, Konrad Malitka, Poland), each containing 500 mg BET. PL was administered in identical-looking white capsules containing cellulose. Participants receiving a daily dose of 2.5 g of BET took three capsules in the morning and two in the evening. The group supplementing with 5.0 g BET per day ingested four capsules in the morning, three in the afternoon, and three in the evening. The capsules were ingested with at least 250 mL of water. The randomized order (BET first or PL first) of the supplementation was double-blinded, for the researcher and the participant. The supplements were prepared by the third party. The doses were not blinded, because we did not want to expose our subjects to excessive amounts of fiber (PL was cellulose), so the number of capsules per day was different in both supplemental groups.

### 2.4. Body composition

Body composition was measured fasted in the morning based on air displacement plethysmography using a Bod Pod (Cosmed, Italy). Once the body density had been determined, the fat mass (FM) and fat-free mass (FFM) were calculated using the Siri equation. Thoracic lung volume was estimated using the Bod Pod software. During measurement, participants wore only a swimsuit and an acrylic swim cap. Repeatability of the BodPod measurements was previously determined as high [12]. Total body water (TBW) was assessed by bioelectric impedance with BodyStat 1500MDD (UK). The recommended measurement conditions were strictly followed during the bioimpedance analysis [13]. Repeatability of the BodyStat 1500MDD measurements was previously determined as good (ICC = 0.96, SEM = 4.5%) [12].

### 2.5. Anaerobic capacity measurement

Anaerobic capacity was assessed using the classic WAnT test on a cycloergometer (Monark 894E, Varberg, Sweden), following the recommendations for such tests proposed by Bar-Or [14]. The test was preceded by a five-minute warm-up period of approximately 50 W power. The test lasted for thirty seconds. External loading was estimated individually at 7.5% body weight. The recorded results were analyzed using Monark Anaerobic Test Software (ver. 3.0.1, 2009, Varberg, Sweden). The following power data were analyzed: peak power (PP), average power (AP), and minimum power (MP), as well as power in each second of the test and mean power during five-second and ten-second intervals.

### 2.6. CF performance

Twenty minutes after the WAnT test, CF performance was measured using the FGB workout, which has been previously described [12,15]. The FGB consists of three rounds of five different multijoint CF exercises including wall ball shots, sumo deadlift high-pulls, box jumps, push presses, and rowing. Participants performed as many repetitions as possible of each exercise for one minute, and then immediately moved to the next exercise until all five exercises were completed. The participants then had a one-minute break between rounds. This test took exactly seventeen minutes to complete (3 rounds × 5 min and 2 breaks × 1 min; each 5-min round consisted of 5 exercises × 1 min). Repetitions were counted if the participant completed the full range of motion required for each exercise. The repeatability of FGB was previously determined to be high (ICC = 0.90, SEM = 6.4%, MDC = 34 reps) [12].

### 2.7. Dietary data

Before each study meeting participants completed a three-day food diary. Participants received detailed instructions on the type of food and drink consumed, time of food consumption, culinary techniques, and recipes (which should be recorded using household measures). Food diaries were then analyzed for nutrient intake using Dieta 6.0 software (National Institute of Public Health (PZH), National Research Institute, Warsaw, Poland).

### 2.8. Blood collection and analysis

Vein blood was collected in the morning of each study meeting in a fasted state by certified personnel. After centrifugation, plasma was stored at −80°C until needed for analysis. Selected hormone concentrations in the plasma were determined using commercially available ELISA kits: EIA1887 for cortisol, EIA4140 for insulin-like growth factor 1 (IGF-1), and EIA1787 for testosterone (DRG International, Springfield, NJ, USA). Absorbance was determined with a microplate reader and associated software (Infinite Pro 200 with i-control, Tecan, Austria).

### 2.9. MTHFR genotyping

Blood samples for *MTHFR* genotyping (rs180113) were taken at the first study meeting in the morning. DNA was isolated from blood lymphocytes using a standard kit (NucleoSpin Blood, Mercherey-Nagel, Germany). Genotyping was performed using TaqMan probes (single-tube assays; Thermo Scientific, USA, assay ID C___1202883_20) on a LightCycler 480 instrument (Roche Diagnostics, Switzerland).

### 2.10. Statistical analysis

Data normality was evaluated with the Shapiro – Wilk test. A series of within/between-subject repeated measure analyses of variance (ANOVA) in a general linear model was used to compare measurements of performance, muscle power, body composition, and hormone concentrations. The within factors were treatment (BET and PL) and time (before or after supplementation). The between factors were *MTHFR* genotype groups (T-allele carriers and people with CC genotype; only the dominant model of inheritance was considered) and BET dose (2.5 and 5.0 g/d). For all measured variables, the estimated sphericity was tested with Mauchly’s W, and the Greenhouse – Geisser correction was used when necessary. Absolute changes (after BET (BET_post_) – before BET (BET_pre_) (ΔBET) and after PL (PL_post_) – before PL (PL_pre_) (ΔPL)) were used to examine the differences in response to supplementation using the dependent *t*-test. All analyses were performed using SPSS Version 22 (IBM, USA) and an alpha level of <0.05 was set *a priori*.

## 3. Results

Out of 84 participants screened for eligibility, 31 were excluded (22 did not meet inclusion criteria, 19 declined to participate). Fifty-five were randomized and allocated to 2.5 g/d BET and 5.0 g/d BET. Forty-three participants attended all four study meetings and were analyzed. Eleven participants dropped out of the study for the following reasons: injury (*n* = 1), COVID-19 isolation or quarantine (*n* = 6), other infection (*n* = 1), moving to a different city (*n* = 1), urgent business trip (*n* = 1), reason unknown (*n* = 1). Genotyping showed 20 participants were T-allele carriers and 23 were CC-allele homozygotes. No side effects were reported throughout the study. There were no differences between groups (2.5 g/d and 5.0 g/d) at baseline (Table 1). No differences were also observed in nutrient intake during the intervention (Table 2).

**Table 1.** Baseline characteristics of participants.

	2.5 g/d group	5.0 g/d group	p-value (T-test)
Age (years)	34.1 ± 6.0	34.2 ± 6.4	.964
Trainings (units per week)	4.3 ± 1.0	4.3 ± 1.1	.835
CrossFit experience (years)	4.1 ± 2.0	3.6 ± 2.4	.997
Height (cm)	179 ± 7	178 ± 5	.587
Body mass (kg)	81.9 ± 1.0	82.7 ± 9.0	.765
Fat mass (%)	18.4 ± 7.6	16.6 ± 5.5	.391
Total body water (%)	55.2 ± 3.7	56.1 ± 4.5	.465

**Table 2.** Participant dietary macronutrient for all study meetings.

	BET_pre_ (mean±SD)	BET_post_ (mean±SD)	PL_pre_ (mean±SD)	PL_post_ (mean±SD)	ANOVA
Carbohydrates (g/kg)	4.42 ± .89	4.36 ± .90	4.45 ± .78	4.43 ± .94	Time × treatment: p = 0.832 η^2^ = 0.024
Protein (g/kg)	1.70 ± .33	1.76 ± .38	1.74 ± .38	1.80 ± .35	Time × treatment: p = 0.255 η^2^ = 0.108
Fat (g/kg)	1.17 ± .34	1.18 ± .31	1.15 ± .35	1.16 ± .33	Time × treatment: p = 0.967 η^2^ = 0.007

BET_post_, after betaine; BET_pre_, before betaine; PL_post_, after placebo; PL_pre_, before placebo.

There were no significant time × treatment interactions for body mass (BM), FM, FFM, or TBW (Table 3).

**Table 3.** Effects of betaine supplementation on body mass and composition.

		BET_pre_ (mean±SD)	BET_post_ (mean±SD)	PL_pre_ (mean±SD)	PL_post_ (mean±SD)	ANOVA
BM (kg)	All	82.2 ± 8.9	82.1 ± 9.0	82.2 ± 9.4	82.1 ± 9.1	Time × treatment: p = 0.881 η^2^ = 0.001
	2.5 g/d BET	81.6 ± 9.3	81.4 ± 9.4	81.9 ± 9.9	81.6 ± 9.1	Time × treatment × dose:
	5.0 g/d BET	82.9 ± 8.5	83.0 ± 8.7	82.5 ± 9.1	82.8 ± 9.3	p = 0.498 η ^2^ = 0.012
FM (kg)	All	14.3 ± 6.6	13.9 ± 6.3	14.4 ± 6.4	14.1 ± 6.3	Time × treatment: p = 0.912 η^2^ = 0.000
	2.5 g/d BET	15.0 ± 7.4	14.2 ± 6.9	15.1 ± 6.7	14.5 ± 6.5	Time × treatment × dose:
	5.0 g/d BET	13.5 ± 5.5	13.5 ± 5.7	13.6 ± 6.0	13.5 ± 6.2	p = 0.565 η^2^ = 0.009
FFM (kg)	All	67.9 ± 7.1	68.2 ± 6.9	67.7 ± 6.8	68.0 ± 6.8	Time × treatment: p = 0.997 η^2^ = 0.000
	2.5 g/d BET	66.6 ± 7.4	67.2 ± 7.0	66.8 ± 6.9	67.0 ± 6.8	Time × treatment × dose:
	5.0 g/d BET	69.4 ± 6.7	69.5 ± 6.8	68.9 ± 6.7	69.3 ± 6.9	p = 0.314 η^2^ = 0.026
TBW (L)	All	45.5 ± 4.6	45.5 ± 4.5	45.5 ± 4.7	45.3 ± 4.5	Time × treatment: p = 0.683 η^2^ = 0.004
	2.5 g/d BET	45.0 ± 5.2	45.0 ± 4.9	45.1 ± 5.3	44.8 ± 4.9	Time × treatment × dose:
	5.0 g/d BET	46.2 ± 4.6	46.1 ± 4.0	46.0 ± 3.9	45.9 ± 4.1	p = 0.823 η^2^ = 0.001

All *n* = 43, 2.5 g/d *n* = 24, 5.0 g/d *n* = 19; BETpost, after betaine; BETpre, before betaine; BM, body mass; FFM, fat-free mass; FM, fat mass; PLpre, before placebo; PLpost, after placebo; SD, standard deviation; TBW, total body water.

**Figure 1.**
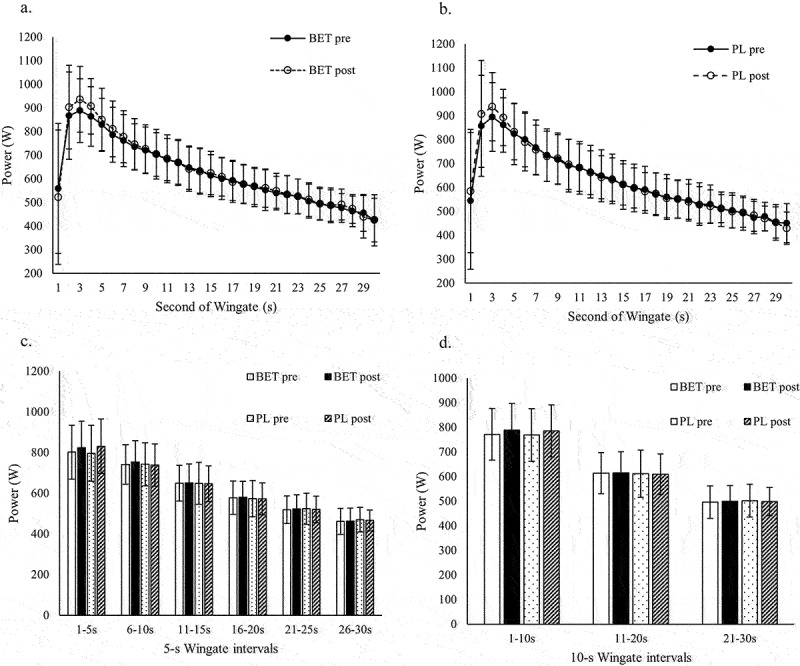
Effects of betaine supplementation on WAnT. a. Effects of betaine on WAnT power, second by second. b. Effects of placebo on WAnT power second by second. c. Effects of betaine on WAnT power in 5-s intervals. d. Effects of betaine on WAnT power in 10-s intervals. BET post, after betaine; BET pre:,before betaine, PL post, after placebo; PL pre, before placebo.

There were no significant time × treatment interactions for PP, AP, or MP (Table 4), or for each second of the WAnT and 5-s and 10-s intervals (Figure 1).

**Table 4.** Effects of betaine supplementation on WAnT power.

		BETpre (mean±SD)	BETpost (mean±SD)	PLpre (mean±SD)	PLpost (mean±SD)	ANOVA
PP (W)	All	963 ± 215	1016 ± 163	981 ± 157	1011 ± 164	Time × treatment: p = 0.423 η^2^ = 0.017
	2.5 g/d BET	936 ± 184	976 ± 132	967 ± 148	981 ± 149	Time × treatment × dose:
	5.0 g/d BET	997 ± 225	1066 ± 187	998 ± 170	1049 ± 176	p = 0.841 η^2^ = 0.001
AP (W)	All	645 ± 94	660 ± 87	654 ± 91	657 ± 86	Time × treatment: p = 0.189 η^2^ = 0.044
	2.5 g/d BET	633 ± 87	638 ± 87	639 ± 95	635 ± 88	Time × treatment × dose:
	5.0 g/d BET	660 ± 102	687 ± 81	673 ± 84	684 ± 79	p = 0.699 η^2^ = 0.004
MP (W)	All	410 ± 67	405 ± 64	414 ± 56	419 ± 68	Time × treatment: p = 0.499 η^2^ = 0.012
	2.5 g/d BET	397 ± 53	397 ± 61	397 ± 55	412 ± 85	Time × treatment × dose:
	5.0 g/d BET	427 ± 80	416 ± 71	435 ± 51	427 ± 40	p = 0.702 η^2^ = 0.004

All *n* = 43, 2.5 g/d *n* = 24, 5.0 g/d *n* = 19; AP, average power; BET_post_, after betaine; BET_pre_, before betaine; MP, minimum power; PL_pre_, before placebo; PL_post_, after placebo; PP, peak power; SD, standard deviation.

The interaction with *MTHFR* was not significant for any outcome measure (Suppl. Table S1 and S2).

BET supplementation significantly increased the number of repetitions independently of the dose (BET_pre_ vs BET_post_) of sumo deadlift high pulls in Round 2 (+1.1 ± 2.7 reps, + 9.5 ± 19.9%) and Round 3 (+1.3 ± 3.2 reps, + 9.4 ± 16.4%); of box jumps in Round 2 (+1.2 ± 2.8 reps, + 14.4 ± 31.3%); and of push presses in Round 1 (+2.3 ± 2.5 reps, 16.8 ± 19.4%), Round 2 (+2.0 ± 3.8 reps, + 18.3 ± 29.7%) and Round 3 (+2.4 ± 3.7 reps, + 16.1 ± 18.7%) in all participants (Table 5). There were no significant interactions with *MTHFR* genotype or BET dose in any of the FGB exercises (Suppl. Table S2 and 3).

**Table 5.** Effects of betaine supplementation on each Fight Gone Bad exercise.

		BETpre (mean±SD)	BETpost (mean±SD)	PLpre (mean±SD)	PLpost (mean±SD)	ANOVA *Time × treatment*
*Wall ball*	Round 1	30.0 ± 3.7	30.2 ± 4.2	30.5 ± 4.4	30.7 ± 5.1	p = 0.819 η^2^ = 0.001
	Round 2	23.0 ± 4.8	24.5 ± 4.4	23.4 ± 4.9	24.7 ± 5.2	p = 0.728 η^2^ = 0.003
	Round 3	21.9 ± 4.6	22.4 ± 4.8	22.1 ± 5.2	22.4 ± 5.5	p = 0.388 η^2^ = 0.020
*Sumo deadlift high pull*	Round 1	20.0 ± 4.3	21.0 ± 5.7	20.3 ± 4.8	20.0 ± 4.8	p = 0.101 η^2^ = 0.068
	Round 2	15.1 ± 4.4^a^	16.3 ± 4.5^b^	15.7 ± 4.6^ab^	15.9 ± 4.3^ab^	p = 0.043 η^2^ = 0.104
	Round 3	14.2 ± 4.2^a^	15.2 ± 4.0^b^	14.6 ± 4.5^ab^	14.5 ± 4.0^ab^	p = 0.049 η^2^ = 0.101
*Box jump*	Round 1	15.9 ± 4.3	16.4 ± 3.7	16.2 ± 4.6	16.3 ± 4.1	p = 0.236 η^2^ = 0.036
	Round 2	13.2 ± 4.7^a^	14.5 ± 4.3^b^	13.7 ± 4.1^ab^	13.7 ± 4.5^a^	p = 0.010 η^2^ = 0.161
	Round 3	12.5 ± 4.4	13.1 ± 4.3	13.1 ± 4.0	13.4 ± 4.5	p = 0.395 η^2^ = 0.019
*Push press*	Round 1	17.2 ± 5.7^a^	19.6 ± 5.7^b^	19.4 ± 7.0^b^	19.2 ± 6.1^b^	p < 0.001 η^2^ = 0.261
	Round 2	14.9 ± 5.7^a^	16.9 ± 5.8^b^	15.9 ± 6.0^ab^	16.2 ± 5.8^ab^	p = 0.013 η^2^ = 0.153
	Round 3	15.4 ± 6.2^a^	17.5 ± 6.5^b^	16.0 ± 6.6^b^	15.9 ± 5.3^ab^	p = 0.003 η^2^ = 0.199
*Rowing*	Round 1	14.2 ± 3.5	15.3 ± 3.2	14.8 ± 3.0	15.1 ± 2.7	p = 0.128 η^2^ = 0.045
	Round 2	12.4 ± 3.4	13.1 ± 2.9	13.1 ± 3.5	13.6 ± 2.7	p = 0.535 η^2^ = 0.010
	Round 3	14.3 ± 2.8	14.7 ± 2.7	14.4 ± 3.0	14.9 ± 3.1	p = 0.689 η^2^ = 0.004
*FGB Total score*		252.4 ± 52.4^a^	270.6 ± 46.7^b^	263.1 ± 53.4^ab^	262.4 ± 56.6^ab^	p < 0.001 η^2^ = 0.313

BETpost, after betaine; BETpre, before betaine; MTHFR, methyltetrahydrofolate reductase; PLpre, before placebo; PLpost, after placebo; SD, standard deviation.

**Figure 2.**
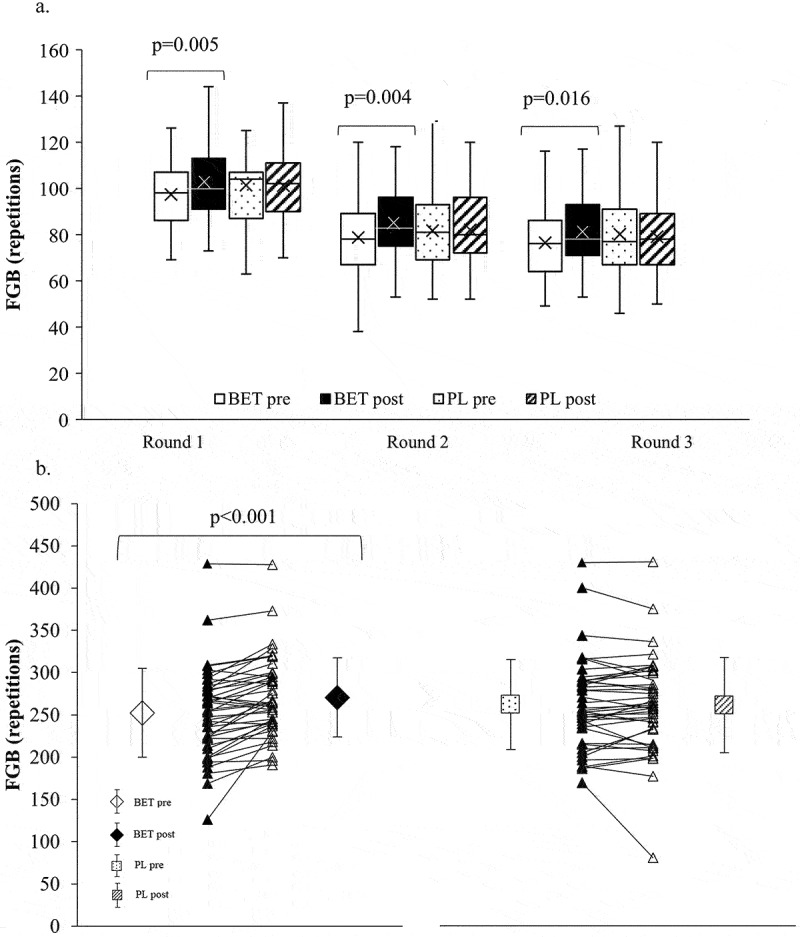
Effects of betaine supplementation on FGB performance. A. Effects of betaine on each round of FGB. B. Effects of betaine on FGB total. BET post, after betaine; BET pre, before betaine; FGB, Fight Gone Bad; PL post, after placebo; PL pre, before placebo.

The total number of repetitions in all five exercises was significantly increased after BET treatment as compared to before BET in each round: the increase in Round 1 was: +5.1 ± 7.6 reps, +5.7 ± 8.0% (*p* = 0.005, η^2^ = 0.188); in Round 2: +6.5 ± 8.3 reps, +10.1 ± 14.9% (*p* = 0.004, η^2^ = 0.200); and in Round 3: +5.6 ± 13.0 reps, 7.3 ± 17.1% (*p* = 0.016, η^2^ = 0.142) (Figure 2). There were no significant differences in the three FGB rounds with PL (Round 1: 0.0 ± 8.4 reps, +0.5 ± 8.5% (*p* = 0.885); Round 2: +0.4 ± 10.7 reps, +0.2 ± 18.2% (*p* = 0.122); Round 3: −1.0 ± 12.4 reps, −0.8 ± 20.5% (*p* = 0.907)). The total of all exercises in all rounds (the FGB total) also saw significant improvement after BET (+18.2 ± 21.4 reps, +8.7 ± 13.6%, *p* < 0.001, η^2^ = 0.313) and no difference after PL (- 0.7 ± 19.9 reps, −0.4 ± 10.0%, *p* = 0.128). ΔBET (+18.8 ± 21.4 reps) in the FGB total was also significantly higher than ΔPL (- 0.7 ± 19.9 reps) (*p* < 0.001). However, no significant interaction with *MTHFR* (time x treatment x *MTHFR* for Round 1 *p* = 0.823, Round 2 *p* = 0.100, Round 3 *p* = 0.621 and FBG total *p* = 0.917) or dose (time x treatment x dose for Round 1 *p* = 0.730, Round 2 *p* = 0.855, Round 3 *p* = 0.924 and FBG total *p* = 0.624) was found in FGB scores.

Testosterone concentration increased significantly after BET supplementation (+0.29 ± 0.67 ng/mL, +7.0 ± 15.4%) with no difference after PL (- 0.02 + 0.69 ng/mL, −1.6 + 19. %, *p* = 0.884) (Table 6). The ΔBET (+0.29 ± 0.67 ng/mL) in testosterone concentration was also significantly higher than testosterone ΔPL (- 0.02 ± 0.69 ng/mL) (*p* = 0.040). However, there were no significant time × treatment interactions for cortisol or IGF-1 concentrations (Table 6). There was also no significant interaction with *MTHFR* genotype or BET dose.

**Table 6.** Effects of betaine supplementation on testosterone, insulin-like growth factor 1, and cortisol concentrations.

		BETpre (mean±SD)	BETpost (mean±SD)	PLpre (mean±SD)	PLpost (mean±SD)	ANOVA
Testosterone(ng/mL)	All	4.77 ± 1.87^a^	5.06 ± 2.02^b^	4.84 ± 1.82^ab^	4.82 ± 1.84^ab^	Treatment × time: p = 0.046 η^2^ = 0.098
	2.5 g/d BET	4.70 ± 2.10	4.98 ± 2.18	4.80 ± 1.90	4.75 ± 2.00	Time × treatment × dose:
	5.0 g/d BET	4.88 ± 1.58	5.17 ± 1.86	4.90 ± 1.77	4.90 ± 1.66	p = 0.928 η^2^ = 0.000
Cortisol (ng/mL)	All	263 ± 130	265 ± 144	270 ± 148	270 ± 155	Treatment × time: p = 0.941 η^2^ = 0.000
	2.5 g/d BET	254 ± 125	249 ± 125	278 ± 154	272 ± 166	Time × treatment × dose:
	5.0 g/d BET	274 ± 140	284 ± 167	260 ± 143	267 ± 144	p = 0.881 η^2^ = 0.001
IGF-1 (ng/mL)	All	170 ± 85	179 ± 97	193 ± 101	180 ± 100	Treatment × time: p = 0.198 η^2^ = 0.044
	2.5 g/d BET	173 ± 85	167 ± 98	191 ± 97	182 ± 100	Time × treatment × dose:
	5.0 g/d BET	166 ± 88	174 ± 99	195 ± 109	178 ± 103	p = 0.420 η^2^ = 0.017

All *n* = 43, 2.5 g/d *n* = 24, 5.0 g/d *n* = 19; BET_post_, after betaine; BET_pre_, before betaine; IGF-1, insulin-like growth factor 1; PL_pre_, before placebo; PL_post_, after placebo; SD, standard deviation.

## 4. Discussion

Our results showed that BET significantly improved CF performance, which was the main outcome of the study. Specifically, BET increased the number of repetitions in all three rounds of FGB separately and also for total FGB score. There is only one other study of BET supplementation in CF [16]. Unlike our study, the authors did not observe there to be an effect of six-week BET supplementation on any performance index. However, there are several differences between the studies: Firstly, Moro et al. [16] used completely different performance tests – no more than three repetitions of back squat, a two-kilometer rowing test on an ergometer, and the Bergeron Beep Test, which lasts about 3.0–3.5 min. Comparing our results with theirs is hence difficult. Secondly, the study of Moro et al. used both females and males [16]. This is important, as BET may affect testosterone concentrations, as shown by our study and by that of Nobari et al. [17].

Even though BET improved CF performance, no significant changes were found in muscular power measured as PP, AP, and MP in the 30-s WAnT. Similar conclusions were made by Hoffman et al. [4] who did not find any differences in PP and AP in two WAnTs after seven and fourteen days of BET supplementation (2.5 g/d). In contrast, Pryor et al. [5] found that seven-day BET ingestion (2.5 g/d) improved sprint performance in a series of four 12-s cycling sprints. It seems that the discrepancy in muscular power results may be due to differences in duration of sprinting. In our study and that of Hoffman et al. [4], the sprints lasted for 30 s, while in the study of Pryor et al. [5] they were shorter (12 s). This could result in different energy substrate utilization.

It should be underlined that the mechanism by which BET could have ergogenic potential is still not fully understood. An interesting observation that can be made based on our study is that BET improved 17-min CF test, but had no influence on anaerobic power in 30 s WAnT. This may indicate the possible application of BET. FGB is an exhausting test, in which athletes must complete as many repetitions as possible of each exercise in 1 min and then move right to the next exercise. The score in FGB is reliant on muscular endurance (performing multiple repetitions with low to moderate resistance). It seems plausible that BET may improve the ability of muscles to exert force, consistently and repetitively, over a period of time. Arazi et al. [18] showed previously that BET improved muscle endurance in leg press and bench press. Moreover, it should be noted that FGB and WAnT differ in terms of energy systems engaged in the force production. FGB engages the phosphagen, glycolytic (as demonstrated previously by elevated lactate concentrations [12]), as well as the aerobic system (since glycolytic system cannot be used in isolation for such a long time and aerobic system is also activated during 1 min rest between the rounds). On the other hand, WAnT engages mostly the phosphagen and glycolytic systems. Those differences in energy systems engaged in FGB and WAnT may provide a clue on why BET supplementation improved FGB performance, but not WAnT.

One possible explanation of BET effect on muscular adaptations may be its influence on hormone concentrations. In our study, testosterone concentration increased after BET treatment; this is in agreement with previous studies [17,18]. Testosterone’s main action is masculinization, but it is also an anabolic agent that enhances muscle hypertrophy and strength [19]. Interestingly, BET concentrations were very high in rat testes, with more BET only being found in rat liver and kidneys [20]. In the testis BET may play the role of a methyl donor, since Leydig cells can involve methylation processes that affect testosterone synthesis [21]. In this way, by increasing methylation agents (i.e. S-adenosyl-methionine), BET may influence steroidogenesis. Animal data suggested methylation inhibitors and homocysteine thiolactone interfered with hormone-stimulated testosterone synthesis [22]. BET can also act as a protectant of testicular tissue under stress. BET ameliorated testicular damages triggered by torsion/detorsion in rats [23] and preserved normal concentrations of testosterone and dihydrotestosterone in rats exposed to arsenic, likely due to reduced oxidative damage in Leydig cells [24]. Taken together, the exact mechanism of BET’s influence on testosterone production has not been well-described, but it may be connected to improved methylation status and the protective role of BET in testicular tissue.

The physiological significance of the observed in our study 7% increase in total testosterone may be questioned. Although testosterone acts as an anabolic agent and exogenous testosterone treatment results in muscle hypertrophy [25], the 7% endogenous increase in testosterone concentrations seems to be fairly modest compared to 2.3 fold increase with exogenous androgen administration [26]. We did not observe any increase in FFM with BET supplementation that would correspond with the increased testosterone. It is possible that such small alteration in the testosterone levels over only 3 weeks was not sufficient to induce growth in skeletal muscles. Another limitation is the fact that we measured only total and not free testosterone, which is considered active.

In contrast to the study of Apicella et al. [7], we did not find any significant differences in IGF-1 or cortisol concentrations. Animal studies also showed increased IGF-1 and GH with BET [27,28]. IGF-1 and GH are anabolic hormones that induce muscle protein synthesis, so BET affecting their concentration would act as a potential nutrient increasing the muscle hypertrophy. However, our study and that of Nobari et al. [16] did not show increased IGF-1 or decreased cortisol after BET in humans.

In our study, 3-week BET supplementation did not affect BM, FM, FFM, or TBW. The effects of BET on body composition have previously been measured by several studies, though with conflicting results [6,15,16]. In contrast to our study, Cholewa et al. [6] showed that FM significantly reduced and FFM increased after six weeks of BET supplementation (2.5 g/d) in resistance-trained young males. Later studies did not however show any effect of BET on body composition [15,16]. This is in agreement with our results and with the most recent meta-analysis, which did not show any beneficial effects of BET supplementation on body composition indices (BM, body mass index (BMI), FM, and FFM) [29].

One of our hypotheses was additionally that T-allele carriers in the *MTHFR* gene would benefit more from BET supplementation. This hypothesis was based on the fact that T-allele carriers may need more BET for methyl groups in homocysteine methylation because, as mentioned before, they cannot effectively use folates due to reduced MTHFR activity [10]. However, we did not find any interaction with BET supplementation, *MTHFR* genotype, or any measured outcome. This might be due to the enhanced oxidation of choline to betaine in T-allele carriers, which satisfies the increased demand for betaine [10].

We also did not observe any differences between the two doses used in our study in terms of any outcome, which suggests that 2.5 g/d BET is sufficient to induce CF performance enhancement and increase testosterone concentration.

Our study is not without limitations. First, we did not measure the folate status of our participants; this can have a major impact on the homocysteine methylation process, especially in T-allele carriers. Secondly, the duration of BET supplementation could have been too short to allow observations of any changes in body composition. Thirdly, we did not measure body water distribution and, since BET is an osmoregulator that protects cells from dehydration, supplementation could have an effect on the relationship between intracellular and extracellular water contents. Fourthly, the interpretation of hormonal data is limited by the fact that the measurement of total testosterone does not provide sufficient information. Future studies should also include free testosterone measurement, since it is the active form of the hormone.

In conclusion, three weeks of BET supplementation may increase CF performance measured in the high-intensity FGB test, as well as total testosterone concentration. BET has no effect on body composition, muscle power in WAnT, or cortisol and IGF-1 concentrations. There was no evidence of a difference between dosages (2.5 and 5.0 g/d) and *MTHFR* genotypes in regards to BET supplementation, although smaller differences, which were undetected here, could still exist. Further studies aiming at identifying these differences are warranted.

## Supplementary Material

Supplemental Material
