# Supplementary material for: Betaine supplementation improves CrossFit performance and increases testosterone levels, but has no influence on Wingate power: randomized crossover trial
Source: J Int Soc Sports Nutr. 2023 Jul 6;20(1):2231411. doi: 10.1080/15502783.2023.2231411 (PMC10327519; doi:10.1080/15502783.2023.2231411)
Supplement: Supplemental Material [file RSSN_A_2231411_SM5839.docx]

Suppl. Table 1. The interaction of *MTHFR* genotype with betaine supplementation on body composition, WAnT power and hormones concentrations.

|  |  | **BET_pre_ (mean±SD)** | **BET_post_**  **(mean±SD)** | **PL_pre_**  **(mean±SD)** | **PL_post_**  **(mean±SD)** | **ANOVA** |
| --- | --- | --- | --- | --- | --- | --- |
| **BM (kg)** | **T-allele** | 83.4 ± 8.0 | 83.6 ± 8.1 | 83.0 ± 8.4 | 82.9 ± 8.0 | *Time × treatment × MTHFR:* |
|  | **CC** | 81.1 ± 9.7 | 80.7 ± 9.7 | 81.4 ± 10.3 | 81.4 ± 10.1 | *p = 0.119*  *η ^2^ = 0.061* |
|  |  |  |  |  |  |  |
| **FM (kg)** | **T-allele** | 15.2 ± 7.6 | 14.8 ± 6.8 | 14.9 ± 6.9 | 14.4 ± 6.9 | *Time × treatment × MTHFR:* |
|  | **CC** | 13.5 ± 5.6 | 13.1 ± 5.9 | 14.0 ± 6.0 | 13.8 ± 6.0 | *p = 0.584*  *η^2^ = 0.008* |
|  |  |  |  |  |  |  |
| **FFM (kg)** | **T-allele** | 68.1 ± 8.2 | 68.8 ± 7.8 | 68.1 ± 7.7 | 68.5 ± 7.6 | *Time × treatment × MTHFR:* |
|  | **CC** | 67.6 ± 6.3 | 67.7 ± 6.2 | 67.4 ± 6.1 | 67.6 ± 6.2 | *p = 0.544*  *η^2^ = 0.010* |
|  |  |  |  |  |  |  |
| **TBW (L)** | **T-allele** | 46.3 ± 5.0 | 46.0 ± 4.7 | 46.4 ± 4.8 | 45.8 ± 4.8 | *Time × treatment × MTHFR:* |
|  | **CC** | 44.8 ± 4.2 | 45.0 ± 4.3 | 44.7 ± 4.5 | 44.8 ± 4.3 | *p = 0.675*  *η^2^ = 0.005* |
|  |  |  |  |  |  |  |
| **PP (W)** | **T-allele** | 970 ± 147 | 1016 ± 150 | 973 ± 148 | 1015 ± 160 | *Time × treatment × MTHFR:* |
|  | **CC** | 958 ± 215 | 1016 ± 177 | 988 ± 167 | 1008 ± 170 | *p = 0.470*  *η^2^ = 0.013* |
|  |  |  |  |  |  |  |
| **AP (W)** | **T-allele** | 665 ± 83 | 674 ± 87 | 666 ± 83 | 666 ± 78 | *Time × treatment × MTHFR:* |
|  | **CC** | 628 ± 101 | 647 ± 87 | 643 ± 97 | 650 ± 94 | *p = 0.815*  *η^2^ = 0.001* |
|  |  |  |  |  |  |  |
| **MP (W)** | **T-allele** | 422 ± 57 | 409 ± 64 | 420 ± 41 | 424 ± 34 | *Time × treatment × MTHFR:* |
|  | **CC** | 400 ± 74 | 402 ± 66 | 408 ± 67 | 414 ± 89 | *p = 0.579*  *η^2^ = 0.008* |
|  |  |  |  |  |  |  |
| **Testosterone (ng/mL)** | **T-allele** | 4.74 ± 1.42 | 5.12 ± 1.67 | 5.03 ± 1.39 | 5.05 ± 1.37 | *Time × treatment × MTHFR:* |
|  | **CC** | 4.80 ± 2.68 | 5.01 ± 2.32 | 4.68 ± 2.15 | 4.62 ± 2.18 | *p = 0.677*  *η^2^ = 0.004* |
|  |  |  |  |  |  |  |
| **Cortisol (ng/mL)** | **T-allele** | 217 ± 52 | 215 ± 60 | 218 ± 74 | 219 ± 80 | *Time × treatment × MTHFR:* |
|  | **CC** | 303 ± 162 | 308 ± 180 | 315 ± 180 | 315 ± 189 | *p = 0.883*  *η^2^ = 0.001* |
|  |  |  |  |  |  |  |
| **IGF-1 (ng/mL)** | **T-allele** | 178 ± 101 | 190 ± 125 | 203 ± 112 | 189 ± 115 | *Time × treatment × MTHFR:* |
|  | **CC** | 162 ± 70 | 155 ± 73 | 184 ± 91 | 173 ± 86 | *p = 0.432*  *η^2^ = 0.016* |

T-allele n=20, CC n=23; AP, average power; BET_post_, after betaine; BET_pre_, before betaine; BM, body mass; CC, CC homozygotes in *MTHFR* C677T; FFM, fat-free mass; FM, fat mass; IGF-1, insulin-like growth factor 1; MP, minimum power; MTHFR, methyltetrahydrofolate reductase; PL_pre_, before placebo; PL_post_, after placebo; PP, peak power; SD, standard deviation; T-allele, T-allele carriers (TC and TT) in *MTHFR* C677T; TBW, total body water; WAnT, Wingate anaerobic test.

Suppl. Table 2. The interaction of *MTHFR* genotype with betaine supplementation on number of repetitions of each exercise of Fight Gone Bad.

|  |  | **Round 1** | | **Round 2** | | **Round 3** | |
| --- | --- | --- | --- | --- | --- | --- | --- |
|  |  | **T-allele**  mean±SD | **CC**  mean±SD | **T-allele**  mean±SD | **CC**  mean±SD | **T-allele**  mean±SD | **CC**  mean±SD |
| ***Wall ball*** | **BET_pre_** | 30.1±3.8 | 29.9±3.6 | 24.5±4.8 | 22.1±4.5 | 22.3±5.7 | 21.7±3.4 |
|  | **BET_post_** | 30.9±4.6 | 29.6±3.8 | 25.0±4.9 | 24.3±3.8 | 22.3±5.4 | 23.0±4.2 |
|  | **PL_pre_** | 30.7±4.7 | 30.3±4.3 | 22.9±4.7 | 24.1±4.9 | 22.3±5.3 | 22.4±5.3 |
|  | **PL_post_** | 31.7±5.3 | 29.7±5.5 | 24.3±5.6 | 25.0±4.9 | 21.9±5.7 | 22.7±5.6 |
|  | *Treatment x time x MTHFR* | p=0.651  η^2^=0.005 | | p=0.094  η^2^=0.072 | | p=0.707  η^2^=0.004 | |
| ***Sumo deadlift high pull*** | **BET_pre_** | 19.9±4.4 | 20.1±4.4 | 15.4±4.2 | 15.0±4.7 | 14.4±4.3 | 14.1±4.2 |
|  | **BET_post_** | 21.4±6.0 | 20.7±5.5 | 15.7±4.8 | 16.7±4.5 | 14.6±3.9 | 15.7±4.2 |
|  | **PL_pre_** | 20.1±5.1 | 20.6±4.5 | 15.3±3.9 | 16.4±4.9 | 14.2±4.8 | 15.1±4.4 |
|  | **PL_post_** | 19.8±4.7 | 20.1±5.0 | 15.4±4.3 | 16.2±4.3 | 13.7±3.9 | 15.2±4.2 |
|  | *Treatment x time x MTHFR* | p=0.637  η^2^=0.005 | | p=0.074  η^2^=0.081 | | p=0.534  η^2^=0.011 | |
| ***Box jump*** | **BET_pre_** | 15.3±5.2 | 16.4±3.3 | 13.3±5.4 | 13.3±4.1 | 12.7±5.0 | 11.8±4.6 |
|  | **BET_post_** | 16.0±4.1 | 16.7±3.4 | 14.0±4.9 | 15.1±3.8 | 12.8±4.9 | 13.3±3.7 |
|  | **PL_pre_** | 15.8±5.1 | 16.6±4.1 | 13.6±4.2 | 13.7±4.2 | 12.4±3.0 | 13.6±4.6 |
|  | **PL_post_** | 15.9±4.2 | 16.7±4.0 | 13.1±5.1 | 14.2±4.0 | 12.5±5.7 | 13.7±4.2 |
|  | *Treatment x time x MTHFR* | p=0.650  η^2^=0.005 | | p=0.887  η^2^=0.001 | | p=0.379  η^2^=0.020 | |
| ***Push press*** | **BET_pre_** | 17.0±6.6 | 17.5±5.0 | 14.8±3.9 | 14.7±5.2 | 15.5±6.9 | 14.7±6.4 |
|  | **BET_post_** | 20.0±6.5 | 19.2±5.0 | 17.3±7.6 | 16.7±4.2 | 17.8±7.7 | 17.2±5.3 |
|  | **PL_pre_** | 19.2±7.6 | 19.7±6.7 | 16.6±7.0 | 15.6±5.1 | 17.0±7.5 | 15.0±5.8 |
|  | **PL_post_** | 19.4±7.1 | 19.0±5.3 | 16.7±7.2 | 15.8±4.4 | 15.4±7.1 | 15.6±4.6 |
|  | *Treatment x time x MTHFR* | p=0.715  η^2^=0.003 | | p=0.926  η^2^=0.000 | | p=0.343  η^2^=0.023 | |
| ***Rowing*** | **BET_pre_** | 14.5±2.7 | 14.0±4.2 | 12.8±2.5 | 12.0±4.1 | 14.4±2.7 | 14.3±2.8 |
|  | **BET_post_** | 15.3±2.6 | 15.3±3.8 | 13.4±2.2 | 13.0±3.4 | 15.4±3.0 | 14.4±2.6 |
|  | **PL_pre_** | 15.3±3.1 | 14.4±2.9 | 13.1±4.0 | 13.2±3.2 | 14.2±3.2 | 14.1±2.9 |
|  | **PL_post_** | 15.5±2.7 | 14.8±2.8 | 13.9±2.6 | 13.3±2.7 | 15.1±2.9 | 14.7±3.5 |
|  | *Treatment x time x MTHFR* | p=0.805  η^2^=0.002 | | p=0.325  η^2^=0.026 | | p=0.574  η^2^=0.009 | |

BET_post_, after betaine; BET_pre_, before betaine; CC, CC homozygotes in *MTHFR* C677T; MTHFR, methyltetrahydrofolate reductase; PL_pre_, before placebo; PL_post_, after placebo; SD, standard deviation; T-allele, T-allele carriers (TC and TT) in *MTHFR* C677T.

Suppl. Table 3. The interaction of betaine’s dose with betaine supplementation on number of repetitions of each exercise of Fight Gone Bad.

|  |  | **Round 1** | | **Round 2** | | **Round 3** | |
| --- | --- | --- | --- | --- | --- | --- | --- |
|  |  | **2.5 g/d**  mean±SD | **5 g/d**  mean±SD | **2.5 g/d**  mean±SD | **5 g/d**  mean±SD | **2.5 g/d**  mean±SD | **5 g/d**  mean±SD |
| ***Wall ball*** | **BET_pre_** | 30.2±3.6 | 30.0±3.7 | 23.6±4.8 | 22.7±4.8 | 21.9±4.8 | 22.1±4.4 |
|  | **BET_post_** | 30.5±4.4 | 29.8±3.9 | 25.0±4.5 | 24.2±4.0 | 22.7±4.9 | 22.7±4.7 |
|  | **PL_pre_** | 30.3±5.2 | 30.7±3.4 | 23.3±5.3 | 23.9±4.3 | 22.0±5.6 | 22.7±4.8 |
|  | **PL_post_** | 31.3±5.3 | 29.9±4.8 | 24.5±5.9 | 24.9±4.2 | 22.1±6.3 | 22.6±4.6 |
|  | *Treatment x time x dose* | p=0.223  η^2^=0.038 | | p=0.798  η^2^=0.002 | | p=0.991  η^2^=0.000 | |
| ***Sumo deadlift high pull*** | **BET_pre_** | 19.0±4.2 | 21.2±4.4 | 14.4±3.5 | 16.2±5.2 | 13.3±4.1 | 15.4±4.1 |
|  | **BET_post_** | 20.4±6.2 | 21.7±4.9 | 14.9±3.9 | 17.8±5.0 | 14.1±3.7 | 16.6±4.2 |
|  | **PL_pre_** | 19.0±4.7 | 21.9±4.5 | 15.2±4.3 | 16.8±4.6 | 13.8±4.6 | 15.9±4.3 |
|  | **PL_post_** | 19.1±4.7 | 21.1±4.8 | 15.0±3.8 | 16.9±4.6 | 13.3±3.7 | 16.1±4.1 |
|  | *Treatment x time x dose* | p=0.946  η^2^=0.000 | | p=0.413  η^2^=0.018 | | p=0.858  η^2^=0.001 | |
| ***Box jump*** | **BET_pre_** | 16.0±3.3 | 15.8±5.4 | 13.0±3.9 | 13.6±5.6 | 12.0±3.8 | 12.5±5.8 |
|  | **BET_post_** | 16.6±3.4 | 16.2±4.2 | 14.3±3.9 | 15.0±4.7 | 12.8±3.8 | 13.4±4.8 |
|  | **PL_pre_** | 15.8±4.3 | 16.7±5.0 | 13.5±4.2 | 13.9±4.2 | 11.8±2.2 | 14.6±5.0 |
|  | **PL_post_** | 16.3±3.9 | 16.4±4.4 | 13.3±4.5 | 14.2±4.6 | 12.6±5.2 | 13.6±4.6 |
|  | *Treatment x time x dose* | p=0.536  η^2^=0.010 | | p=0.579  η^2^=0.008 | | p=0.345  η^2^=0.023 | |
| ***Push press*** | **BET_pre_** | 16.4±4.8 | 18.3±6.7 | 14.6±5.3 | 15.5±6.2 | 14.4±5.8 | 15.9±7.6 |
|  | **BET_post_** | 18.9±5.3 | 20.4±6.1 | 16.1±5.9 | 18.0±5.9 | 17.3±6.9 | 17.8±6.0 |
|  | **PL_pre_** | 18.2±6.9 | 21.0±7.1 | 15.4±6.0 | 16.8±6.0 | 15.4±6.8 | 16.7±6.4 |
|  | **PL_post_** | 18.2±5.6 | 20.5±6.6 | 15.0±5.2 | 17.7±6.2 | 14.5±5.9 | 16.8±5.6 |
|  | *Treatment x time x dose* | p=0.971  η^2^=0.000 | | p=0.782  η^2^=0.002 | | p=0.258  η^2^=0.033 | |
| ***Rowing*** | **BET_pre_** | 14.0±2.7 | 14.5±4.5 | 12.2±2.6 | 12.6±4.3 | 14.0±2.6 | 14.7±3.0 |
|  | **BET_post_** | 15.5±3.3 | 15.0±3.2 | 13.0±2.6 | 13.4±3.2 | 14.8±2.8 | 14.9±2.9 |
|  | **PL_pre_** | 14.3±2.9 | 15.4±3.2 | 12.7±3.8 | 13.8±3.1 | 14.0±3.2 | 14.4±2.7 |
|  | **PL_post_** | 14.7±2.8 | 15.7±2.6 | 13.2±2.7 | 14.1±2.6 | 14.3±2.8 | 15.6±3.6 |
|  | *Treatment x time x dose* | p=0.349  η^2^=0.023 | | p=0.750  η^2^=0.003 | | p=353  η^2^=0.023 | |

BET_post_, after betaine; BET_pre_, before betaine; PL_pre_, before placebo; PL_post_, after placebo; SD, standard deviation;
